# A role for tumor necrosis factor-α in ischemia and ischemic preconditioning

**DOI:** 10.1186/1742-2094-8-87

**Published:** 2011-08-02

**Authors:** Orla Watters, John J O'Connor

**Affiliations:** 1 UCD School of Biomolecular and Biomedical Science, UCD Conway Institute of Biomolecular and Biomedical Research, University College Dublin, Belfield, Dublin 4, Ireland

**Keywords:** Tumor Necrosis Factor-alpha, ischemia, hippocampus, glutamate, calcium, preconditioning

## Abstract

During cerebral ischemia, elevation of TNF-α and glutamate to pathophysiological levels may induce dysregulation of normal synaptic processes, leading ultimately to cell death. Previous studies have shown that patients subjected to a mild transient ischemic attack within a critical time window prior to a more severe ischemic episode may show attenuation in the clinical severity of the stroke and result in a more positive functional outcome. Studies with organotypic hippocampal cultures and mixed primary hippocampal cultures have shown that prior incubation with low concentrations of glutamate and TNF-α increase the resistance of neurones to a subsequent insult from glutamate, AMPA and NMDA, while co-exposure of TNF-α and for example AMPA may have neuroprotective effects compared to cultures exposed to excitotoxic agents alone. In addition our work has shown that although glutamate and TNF-α pretreatment induces analogous levels of desensitisation of the intracellular calcium dynamics of neurons under resting conditions and in response to acute glutamate stimulation, their downstream signalling pathways involved in this response do not converge. Glutamate and TNF-α would appear to have opposing effects on resting Ca^2+ ^levels which supports the proposal that they have distinct modes of preconditioning.

## Introduction

### Stroke and brain function

As neurons are incapable of storing glucose, they rely on the cardiovascular system and astrocytes to deliver this source of energy. Thus, the depletion of oxygen (hypoxia) and glucose supply to the neuronal tissue during a stroke, will result in inadequate aerobic metabolism and failure of the cells to generate sufficient ATP levels required to meet metabolic demand [1]. Maintenance of Ca^2+ ^homeostasis is lost due to insufficient ATP to fuel extrusion pumps, while the resting membrane potential is also disrupted due to dysfunction of the Na^+^/K^+ ^ATPase pumps, leading to 'anoxic depolarisation' [2]. The resulting ionic imbalance within the neuronal and glial cells manifests in the development of tissue acidosis [3], cytotoxic oedema and ultimately necrosis [4]. The accumulation of cations in the cytosol contribute to transient depolarisation at the nerve terminals, which in turn triggers the activation of voltage-sensitive Na^2+ ^channels, amplifying the accumulation of positive charge within the nerve terminal [5]. This increase in membrane potential will be detected by the voltage sensors on the intracellular domain of voltage-dependent Ca^2+ ^channels (VDCC) causing a large influx of Ca^2+ ^into the terminal promoting vesicular release of neurotransmitters and gliotransmitters such as glutamate and/or TNF-α from neuronal/glial cells, respectively [6], which at pathophysiological levels, induce cellular toxicity [7,8]. Extracellular levels of glutamate and TNF-α have been shown to remain elevated in the infarct region for hours up to days after a stroke, depending on the severity of the cerebral ischemic event [9-11]. Due to the complex nature of cross-communication between neuronal and glial cells, the contribution of glutamate and TNF-α to neurotoxicity during stroke is intricately interlinked, with both cell types responsible for the excessive elevation of these mediators to pathophysiological levels, by paracrine and/or autocrine signalling [12].

### TNF-α receptor expression and signal transduction

TNF-α activity is mediated through activation of its surface receptors, found on both neuronal [13,14] and glial cell populations [15], along with endothelial cells of the cerebral vasculature [16]. Two TNF-α receptors have been identified, the low affinity TNFR1 (p55) and the high affinity TNFR2 (p75) receptor [17]. Although the extracellular domains to the TNFRs have a high degree of homology, their intracellular domains do not [18], which accounts for the complex signal transduction pathways and corresponding proposed antagonistic functions of these two receptor subtypes [17,19]. TNFRs may exist as pre-aggregated membrane receptor complexes, whereby TNF-α binds to its receptors as a homo-trimeric protein [20], or may form hetero-receptor complexes in response to ligand binding [21]. Upon TNF-α binding, conformational change of the receptors may lead to receptor endocytosis and exposure of its intracellular binding sites for many adaptor proteins. However, it is important to note that receptor shedding from the membrane may also occur, which results in the neutralisation of circulating TNF-α by acting as a 'decoy receptor' [22].

TNF-α-receptor-associated factor-2 (TRAF-2) was first identified as a signal transducer molecule for the TNFR2 TNF-α receptor [23]. TRAF-2 can bind directly to the intracellular domain of TNFR2, and switch on numerous signalling cascades which ultimately result in the activation of the the NFκB transcription factor. TRAF-2 associates with receptor interacting protein (RIP) which in turn activates IKβ kinase (IKKβ) to phosphorylate the inhibitory subunit of NFkB (IκB), leading to dissociation of this subunit, freeing the active NFκB transcription factor, which dimerises and translocates to the nucleus to mediate gene transcription [24].

**Figure 1:**
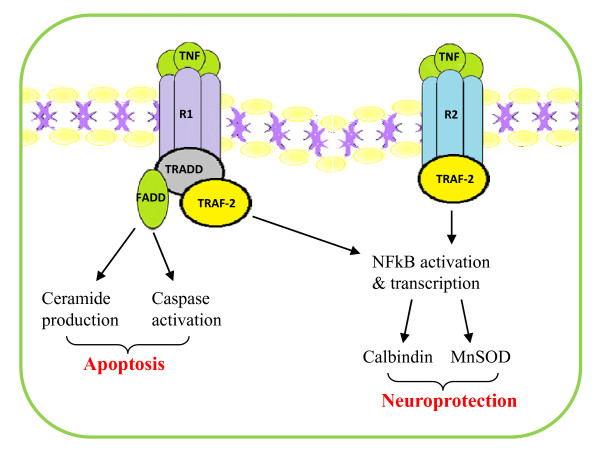
**Summary of TNFR signalling**. TNF-α has two receptors TNFR1 (R1) and TNFR2 (R2) which are co-expressed on both neuronal and glial membranes. Activation of TNFR1 predominantly results in the initiation of caspases involved in apoptosis. For this reason, this receptor is known as the 'death receptor'. However, association of the death domain of this receptor with TRAF-2 can lead to the activation of NFkB gene transcription and upregulation of neuroprotective mediators such as the calcium chelator, calbindin, and the superoxide scavenger, manganese superoxide dismutase (MnSOD). This pathway is also shared with TNFR2 downstream signalling.

Further investigation has revealed a separate role for TRAF-2 in TNFR1 downstream signalling. TNFR1 has been dubbed the 'death receptor' due to the discovery of an intracellular death domain sequence [25]. Upon internalisation of the activated TNFR1 receptor, TNF receptor-associated death domain (TRADD) adaptor molecule associates with its death domain sequence and signals the recruitment of TRAF-2 or RAIDD adaptor protein to its receptor, to initiate downstream activation of cysteine proteases, called caspases, involved in programmed apoptotic cell death. Fas-associated death domain (FADD) can either bind directly to TNFR1 death domain sequence or to the TRAF-2-TRADD complex and induce the auto-cleavage of pro-caspase 8 to its active form [26], while association of TRADD with RAIDD and receptor interacting protein (RIP) activates caspase 2 [27]. See Figure 1 for details.

However, these signalling cascades are not mutually exclusive to the individual receptors [28]. The activation of TRAF-2 by TNFR1 stimulation has also the ability to associate with RIP and initiate NFκB and JNK signalling leading to a neuroprotective response [29]. TNFRs have also been shown to physically interact with insulin receptor substrate-1 (IRS-1) and PI 3-kinase, which may also contribute to the modulation of NFkB gene transcription [30]. It seems that the rate and persistence of NFκB activation differ depending on receptor subtype activated, which may account for the difference in their overall response [19]. A study by Marchetti et al. (2004), using TNFR1^-^/^- ^and TNFR2^-^/^- ^KO mice, found TNF-α had a preconditioning effect on subsequent glutamate insult in TNFR1^-^/^- ^KO mice, whereas TNFR2^-^/^- ^mice were more susceptible to glutamate-induced excitotoxicity. The neurotoxic and neuroprotective effect of TNFR1 and TNFR2, respectively, were further validated using agonistic antibodies for these receptors [19]. This study also found that NFκB activity in response to TNFR1 activation was rapid and transient, while TNFR2 activation resulted in a more slower but persistent response. However, as most cell types express both TNFR1 and TNFR2 their receptor activity are not mutually exclusive. Individual receptor signalling may also be confounded by the fact that these receptors may aggregrate as heter-receptor complexes upon binding of TNF-α [21]. Thus the overlap in their signalling cascades adds to the complexity of the overall effect of TNF-α. It has been postulated that it is the ratio of TNFR1:TNFR2 receptor expression that may govern the overall TNF-α action. These receptors have been shown to be constitutively expressed at different ratios on different cell types, and have been shown to be non-uniformly up-regulated in response to a variety of ligands [15,31].

### Contribution of TNF-α to cerebral ischemia

TNF-α is a well known cytokine involved in the inflammatory response elicited in the region of cerebral ischemia. Indeed, levels of TNF-α may remain elevated in the affected brain tissue for at least 24 h after an ischemic insult [9,10]. However the function of TNF in brain ischemia is controversial. At pathophysiological levels, TNF-α has been shown not only to be involved in necrosis, but also in the regulation of caspases and other apoptotic factors [32,33]. Its initiation of an inflammatory response may result in disruption of the blood-brain barrier, compromising the protective barrier between the brain and the systemic circulation. Thus the infiltration of peripheral inflammatory cells to the affected brain region can occur, exacerbating the overall inflammatory response [34,35]. TNF-α has also been shown to stimulate astrocyte [36] and microglial [15] activation and proliferation in a paracrine and/or autocrine fashion.

In early studies with TNF KO mice Mattson showed clearly that damage to neurons by focal cerebral ischemia and excitotoxic insults was enhanced in TNFR-KO mice, implicating TNF as a neuroprotectant in the brain [37]. These effects were shown to involve increased oxidative stress and supression of injury induced microglial activation. Later studies by the same group implicated both TNFRs, namely p55 and p75 in these effects [38]. More recently Lambertsen et al. (2009) have recently identified a neuroprotective role for microglial dervied TNF in cerebral ischemia an affect shown to be due to TNF-p55R activity [39]. In this work with TNF-p55R KO mice they also reported a reduced microglial population size and interestingly reduced Toll-like receptor 2 expression. The work of Taoufik et al (2008) investigated the molecular mechanisms of the neuroprotective effects of TNF using TNFR1 KO mice using the model of middle cerebral artery occlusion. They found that erythropoietin (originally described as a hematopoietic growth factor) and vascular endothelial growth factor (VEGF) induced neuroprotection against glucose deprivation, NMDA excitotoxicity and oxygen glucose deprivation and that these effects required the presence of TNFR1 [40]. These data together provide stong evidence for that TNF plays a key role in determining the survival of endangered neurons in cerebral ischemia.

Glial cells contain metabotropic type 2 glutamate receptors (mGluR2) on their plasma membrane, which, once activated by excessive glutamate release during cerebral ischemia, results in stimulation of IP_3_-mediated Ca^2+ ^release from intracellular stores and the release of 'gliotransmitters' such as TNF-α, ATP and glutamate itself [41]. TNF-α can act in both an autocrine and a paracrine fashion, stimulating its receptors found on glial and neighbouring neuronal cell membranes. Stimulation of TNFR's present on microglial membranes stimulates the upregulation of glutaminase, an enzyme involved in the conversion of glutamine to its active form, glutamate. Excess glutamate production in the microglia may result in the uncontrolled release of glutamate into the extracellular space via hemichannels present on the microglial membrane [42]. TNF-α can also have negative effects on glutamate uptake and degradation by astrocytes. Activation of the TNFR1-caspase 3 pathway results in the cleavage of EAAT2 at the intracellular C-terminal domain of the glutamate transporter, rendering it inactive [43]. Stimulation of the NFkB pathway in astrocytes by TNF-α, decreases the transcription and expression of EAAT2 on the glial membrane, reducing glutamate uptake from the synaptic cleft [44]. Although this response may favour glial survival by preventing overaccumulation of glutamate in the astrocytes, neuronal survival will be further compromised as a result, due to prolonged excitation of these cells by excessive glutamate in the synaptic cleft [45]. TNF-α stimulation of astrocytes may also result in PKC mediated activation of NFkB transcription, and upregulation of interleukin-6, a potent inflammatory mediator, thus exacerbating the inflammatory response [46].

Activation of the TNFR1 receptor on neurons initiates the caspase cascade involved in apoptosis [8] Badiola et al. (2009) using TNFR KO mice and an OGD model in cortical cultures showed that TNF induced apopotic cell death involves TNFR1-activation of caspase-8 and caspase-3 but not caspase-9 [33]. Neuronal TNFR1-mediated IP_3 _activation may induce the insertion of Ca^2+ ^permeable AMPARs into the post-synaptic density, thus may contribute to Ca^2+^-mediated cell death in this way, during an ischemic insult [47]. Indeed Stellwagen and his colleagues also found that treatment of hippocampal cultures with TNF-α (1 μg/ml) for 15 min was sufficient to induce a reduction in surface GABA_A _receptors in the same cohort of cells (most likely due to endocytosis), reducing the responsiveness of these cells to inhibitory input, which would further exacerbate excitotoxicity during an ischemic insult [47].

**Figure 2:**
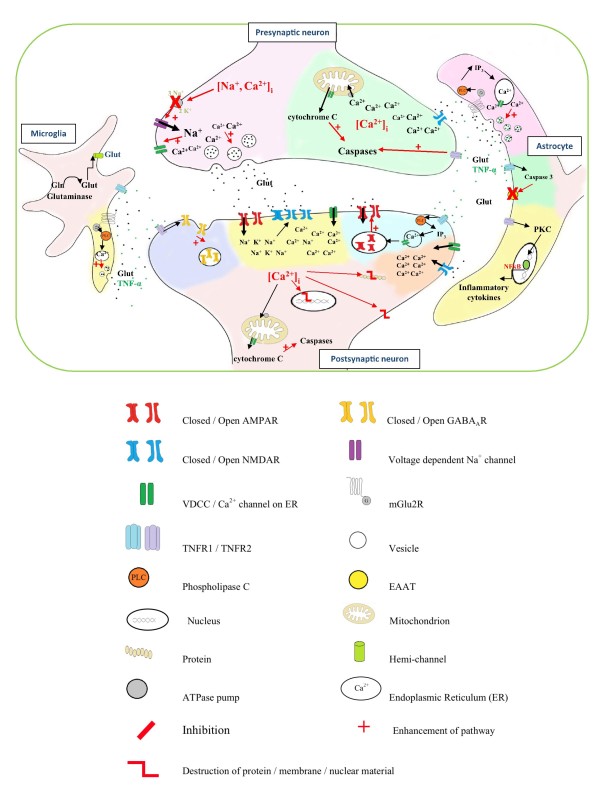
**Summary of the detrimental effects of TNF-α on both neuronal and glial cells during cerebral ischemia**. TNF-α synthesis and release for both neuronal and glial cells may be induced via stimulation of mGlu2R by glutamate. TNF-α may then act in an autocrine and/or paracrine fashion, modulating neuronal and glial signalling. Direct stimulation of the TNFR1-caspase 3 pathway results in inactivation of glial EAAT2s, while activation of the NFkB pathway reduces the synthesis and expression of this glutamate transporter. Upregulation of glutaminase in response to microglial TNFR activation also enhances glutamate synthesis and release, elevating levels of this neurotransmitter in the synapse. TNFR1 signalling may also enhance AMPAR trafficking into the postsynaptic membrane, enhancing the sensitivity of the cells to glutamate. Together, these effects result in the exacerbation of glutamate excitotoxicity during cerebral ischemia.

Together, these studies highlight the vast detrimental effects of TNF-α on both glial and neuronal functioning during cerebral ischemia. TNF-α mediated cell destruction may be mediated directly, via activation of its TNFR and subsequent cell death signalling pathways, or indirectly by enhancing glutamate excitotoxicity. Overall there is convincing data supporting both detrimental and protective effects of TNF in brain ischemia. Figure 2 summarises some of these processes.

### TNF-α and ischemic preconditioning

A milder form of a cerebral ischemic event, known as a transient ischemic attack (TIA), results in a brief disruption of the cerebral blood supply and temporary presentation of stroke-like symptoms. A TIA may occur upon narrowing or temporary blockage of a cerebral artery by a thrombus or an embolism. A TIA is considered a warning sign that a more severe stroke may occur if appropriate measures are not taken to prevent it. Recent studies have shown that a mild TIA within a narrow time window prior to a stroke may enhance the tolerance of the brain to deal with this second insult. In 2000, a study of 2490 stroke patients, found that a TIA must be at least 5 min in duration in order to induce neuroprotection [48]. Conversely, a prolonged TIA (greater than one week) or repetitious TIA's cancel out this neuroprotective effect. This study also found that the neuroprotective effect of a TIA is transient, greatly diminishing within 72 h. Interestingly, a clinical study carried out by Castillo et al. (2003) found that plasma levels of TNF-α remained elevated in patients for up to 72 h after a TIA, and that those who experienced a TIA prior to a stroke within this time window had a better functional outcome than those who did not [49]. Corresponding with the in vivo findings, a study carried out in mixed cortical cultures in vitro found, using brief oxygen and glucose deprivation (OGD) as a model of TIA, that the OGD-induced neuroprotection was also lost within 72 h [50]. The preconditioning effect of a TIA can also been demonstrated in many experimental models of stroke, both in vivo [51-53] and in vitro [54,55].

Elevation of both glutamate and TNF-α may persist for days after a TIA and contribute to the enhancement of cellular defences against a more severe ischemic insult [56-59]. To date, extensive research has been carried out in order to isolate the proposed mechanisms of ischemic tolerance caused by glutamate or TNF-α elevation as part of a TIA. Incubation of primary neuronal cells with TNF-α (100 ng/ml) for 48 h, prior to excitotoxic AMPA or NMDA exposure, significantly reduced the peak Ca^2+ ^response induced by these ionotrophic glutamate receptor agonists [60]. As calcium is a well known mediator of cell death, these findings suggest that TNF-α mediated neuroprotection may be as a result of decreasing conductance of these ionotrophic receptors to Ca^2+^, or reducing their sensitivity to these glutamatergic agents. This study also found that TNF-α induced upregulation of NFkB gene transcription of neuroprotective mediators such as calbindin, a calcium chelator, and manganese superoxide dismutase (MnSOD), a powerful anti-oxidant which contribute to this neuroprotective response [60]. However, it must be noted that these experiments were carried out in primary neuronal cultures, in the absence of the glial cell population.

A study carried out by Bernardino et al. (2005) used organotypic hippocampal cultures to investigate TNF-α mediated preconditioning against subsequent AMPA exposure [61]. Organotypic hippocampal cultures best represent the synaptic morphology of the hippocampus in vivo, as the circuitry of the hippocampus is maintained and the contribution of the glial cell population to the overall response can taken into account [62]. Incubation of organotypic hippocampal cultures with 1 ng/ml TNF-α for 24 h increased the resistance of the CA 1 pyramidal cells to a subsequent AMPA insult, while co-exposure of TNF-α and AMPA also had a neuroprotective effect compared to cultures exposed to AMPA alone [61]. However, this neuroprotective effect was lost when the concentration of TNF-α was increased to 10 ng/ml, while co-exposure exacerbated AMPA-induced toxicity, revealing a concentration-dependent element to the TNF-α mediated response. We have also shown in our laboratories using organotypic hippocampal slices that attenuation in resting Ca^2+ ^activity and Ca^2+ ^related responsiveness of cells within the hippocampus as a result of glutamate or TNF-α pre-exposure, may contribute to the development of ischemic tolerance [63]. We found that inhibition of the p38 MAP kinase pathway with SB 203580 (10 μM), before and during TNF-α exposure resulted in a complete reversal of TNF-α's effect on glutamate-induced Ca^2+ ^influx, 24 h post treatment.

In an *in vivo *study carried out by Sotgiu and colleagues, they showed using a multiple linear regression analysis that there was a positive linear correlation between blood levels of TNF-α (measured 6-20 h post-stroke) and the clinical severity of the cerebral ischemic event, supporting these *in vitro *findings [64]. As a TIA is a much milder form of stroke, it is possible that the lower levels of TNF-α produced during a TIA contribute to induction of neuroprotective mechanisms, while more pathophysiological levels of TNF-α reached during stroke, exacerbate glutamate-mediated excitotoxicity and cell death. Coinciding with this hypothesis, it is possible that the mild elevation of cell death signalling molecules during ischemic preconditioning, such as ceramide and ROS, may trigger the upregulation of neuroprotective mediators, preparing cellular defences against a subsequent more severe ischemic insult [55,65]. Stimulation of neuronal TNFR1 receptors during an initial ischemic preconditioning event has been shown to stimulate increased gene transcription and protein expression of EAAT3 on the neuronal membrane, thus priming the neurons to mop up excess glutamate more rapidly and efficiently from the synaptic cleft, reducing the extent of glutamate excitotoxicity which may be induced by a more severe ischemic insult [66].

The contribution of mild acute glutamate elevation during a TIA to the development of ischemic tolerance has also been extensively studied in vitro. During an ischemic event, oxygen and glucose depletion induces neuronal dysfunction and uncontrolled excessive glutamate release. Oxygen and glucose deprivation (OGD) can be induced in vitro by incubating the cultures in an anaerobic chamber with a gas composition of up to 95% N_2 _and 5% CO_2_, while replacing the culture media with one which is glucose-free. These conditions were found to mimic those occurring in vivo, by inducing both Ca^2+^-dependent and Ca^2+^-independent cell death pathways in both neuronal and glial cell populations [67]. In some cases 2-deoxy-glucose may be added to the glucose free media to inhibit the glycolysis, thus preventing the production of endogenous glucose. In accordance with data obtained from in vivo studies, a short exposure of cultured cells to OGD (10-30 min) mimics the events of a TIA, inducing neuroprotection against a subsequent more severe insult [50]. As OGD induces endogenous glutamate elevation, the increased activity of this excitatory neurotransmitter at its receptors was hypothesised as one of the contributing factors to the development of ischemic tolerance. Indeed, Grabb and Choi (1999) discovered that exposure of the cultures with brief exposure to a mild concentration of glutamate (10-30 μM, 30 min) induced a similar level of neuroprotection against a more prolonged OGD insult applied 24 h later. This effect was also replicated using sublethal NMDA exposure (5-10 μM, 30 min) as a preconditioning agent, while inhibition of NMDAR's during OGD abolished the neuroprotective response, suggesting that glutamate-mediated neuroprotection as a result of NMDAR activation [50]. Activation of NMDAR's during glutamate preconditioning has been shown to induce Ca^2+^-mediated activation of various signalling pathways involved in upregulation neuroprotective mediators [58,68]. Pharmacological dissection of the signalling cascades known to the activated by Ca^2+^, using a variety of antagonists revealed that Ca^2+^-mediated activation CaMK-II which in turn phosphorylates CREB, inducing CRE-mediated gene transcription of Bcl-2, a protein known to suppress apoptosis. This effect was further validated in vivo using temporary middle cerebral artery occlusion (mCAO) to induce TIA-like elevation in glutamate transmission [68]. Ischemic tolerance induced by OGD may also involve activation of other glutamate receptor types such as the ionotrophic AMPAR and the metabotrophic receptor mGluR1, as antagonism of these receptors was found to attenuate OGD-mediated preconditioning to a subsequent severe OGD insult, in organotypic hippocampal cultures [69].

It is important to note that TNF-α and glutamate share numerous downstream signalling pathways such as CREB, NFkB and MAPK, thus their contribution to the development of ischemic tolerance during a TIA may overlap [59,68,70-73]. Indeed, glutamate itself can stimulate the release of pro-inflammatory cytokines such as TNF-α. Keeping this in mind, it is possible that application of exogenous glutamate may have a knock-on TNF-α mediated effect, adding a layer of complexity to the interpretation of experimental findings [74].

## Conclusions

Like many neurological disorders an imbalance in neurotransmission and inflammatory responses within the brain may negatively affect neuronal function, and in more severe cases may result in cell death. Excessive glutamate signalling during cerebral ischemia has been implicated in the development of excitotoxicity, while hightened TNF-α signalling mediates an inflammatory response which is known to exacerbate this detrimental glutamate response. However, mild elevation of glutamate and TNF-α during an acute TIA has highlighted their role as preconditioning stimuli, inducing an endogenous neuroprotective response against subsequent ischemic insults. Numerous pathways and effector molecules are involved in this enhanced tolerance, ultimately resulting in a more stringent regulation of neuronal signalling and calcium responses to excessive glutamate stimulation, induced under conditions of cerebral ischemia.

## List of Abbreviations

(AMPAR): 2-amino-3-(5-methyl-3-oxo-1,2- oxazol-4-yl) propanoic acid receptor; (ATP): adenosine tri-phosphate; (ATPase): adenosine tri-phosphatase; (Bcl-2): B cell lymphoma 2; (Ca^2+^): calcium; (CAM-KII): calcium/calmodulin -dependent protein kinase 2; (CREB): cAMP response element binding protein; (EAAT): excitatory amino acid transporter; (FADD): fas-associated death domain; (GD): glucose deprivation; (GABA): γ-aminobutyric acid; (IkB): inhibitor of kB; (IKKβ): inhibitor of nuclear factor kappa-B kinase subunit beta; (IP_3_): inositol triphosphate; (IRS): insulin receptor substrate; (JNK): c-Jun N-terminal kinase; (KO): knock-out; (MAPK): mitogen-activated protein kinase; (mCAO): middle cerebral artery occlusion; (mGluR): metabotrophic glutamate receptor; (MnSOD): manganese superoxide dismutase; (NFkB): nuclear factor kappa B; (NMDA): N-Methyl-D-aspartic acid; (OGD): oxygen and glucose deprivation; (PI-3): phosphoinositide- 3; (RAIDD): RIP-associated ICH-1 homologous protein with a death domain; (RIP): receptor interacting protein; (SB203580): 4-[5-(4-Fluorophenyl)-2-[4-(methylsulfonyl)phenyl]-1*H*-imidazol-4-yl]pyridine; (TIA): transient ischemic attack; (TNF-α): tumour necrosis factor-alpha; (TNFR): tumour necrosis factor-alpha receptor; (TRAF): TNF-α receptor-associated factor; (TRADD): TNF-α receptor-associated death domain; (VDCC): voltage-dependent calcium channel.

## Competing interests

The authors declare that they have no competing interests.

## Authors' contributions

OW participated in the design of the study, carried out the organotypic and primary hippocampal culture studies referred to in the manuscript, performed the statistical analysis and drafted the manuscript. JOC conceived the study, and participated in its design and coordination and helped to draft the manuscript. Both authors read and approved the final manuscript.
